# Introduction of a Capillary Gel Electrophoresis-Based Workflow for Biotherapeutics Characterization: Size, Charge, and *N-*Glycosylation Variant Analysis of Bamlanivimab, an Anti-SARS-CoV-2 Product

**DOI:** 10.3389/fbioe.2022.839374

**Published:** 2022-03-08

**Authors:** Miklos Szabo, Daniel Sarkozy, Marton Szigeti, Robert Farsang, Zsofia Kardos, Adam Kozma, Eszter Csanky, Doo Soo Chung, Zoltan Szekanecz, Andras Guttman

**Affiliations:** ^1^ Borsod Academic County Hospital, Miskolc, Hungary; ^2^ Horváth Csaba Memorial Laboratory of Bioseparation Sciences, Research Center for Molecular Medicine, Faculty of Medicine, Doctoral School of Molecular Medicine, University of Debrecen, Debrecen, Hungary; ^3^ Translational Glycomics Group, Research Institute for Biomolecular and Chemical Engineering, University of Pannonia, Veszprém, Hungary; ^4^ Department of Chemistry, Seoul National University, Seoul, South Korea; ^5^ Department of Rheumatology, Faculty of Medicine, University of Debrecen, Debrecen, Hungary

**Keywords:** capillary electrophoresis, bamlanivimab, anti-SARS-CoV-2, product characterization, N-glycans

## Abstract

Coronavirus Disease 2019 (COVID-19) is a major public health problem worldwide with 5–10% hospitalization and 2–3% global mortality rates at the time of this publication. The disease is caused by a betacoronavirus called Severe Acute Respiratory Syndrome Coronavirus 2 (SARS-CoV-2). The receptor-binding domain (RBD) of the Spike protein expressed on the surface of the virus plays a key role in the viral entry into the host cell *via* the angiotensin*-*converting enzyme 2 receptor. Neutralizing monoclonal antibodies having the RBD as a target have the ability to inhibit angiotensin-converting enzyme 2 (ACE2) receptor binding, therefore, prevent SARS-CoV-2 infection, represent a promising pharmacological strategy. Bamlanivimab is the first anti-spike neutralizing monoclonal antibody, which got an emergency use authorization from the FDA for COVID-19 treatment. Albeit, bamlanivimab is primarily a neutralizing mAb, some of its effector function related activity was also emphasized. The effector function of antibody therapeutics is greatly affected by their *N-*linked carbohydrates at the conserved Fc region, possibly influenced by the manufacturing process. Various capillary gel electrophoresis methods are widely accepted in the biopharmaceutical industry for the characterization of therapeutic antibodies. In this paper we introduce a capillary gel electrophoresis based workflow for *1*) size heterogeneity analysis to determine the presence/absence of the non*-*glycosylated heavy chain (NGHC) fragment (SDS-CGE); *2*) capillary gel isoelectric focusing for possible *N*-glycosylation mediated charge heterogeneity determination, e.g., for excess sialylation and finally, *3*) capillary gel electrophoresis for *N-*glycosylation profiling and sequencing. Our results have shown the presence of negligible amount of non-glycosylated heavy chain (NGHC) while 25% acidic charge variants were detected. Comprehensive *N*-glycosylation characterization revealed the occurrence of approximately 8.2% core-afucosylated complex and 17% galactosylated *N-*linked oligosaccharides, suggesting the possible existence of antibody dependent cell mediated cytotoxicity (ADCC) effector function in addition to the generally considered neutralizing effect of this particular therapeutic antibody molecule.

## Introduction

Recently emerging viral diseases have become a significant public threat around the world. The coronavirus pandemic (COVID-19) currently represents a major public health problem worldwide since the outbreak in 2019. At the time of this publication, 5–10% of patients are hospitalized, while the mortality rate is around 2–3% globally (Salzberger et al., 2021). Severe illness typically occurs approximately 1 week after the onset of symptoms and can rapidly progress from the mild stage. The risk factors for severe COVID-19 include being male, age >65, and with comorbidities such as cardiovascular disease, lung disease, hypertension, diabetes, or obesity (Wu C. et al., 2020; Williamson et al., 2020). The most common cause of fatal outcome is pneumonia and consequent respiratory failure. The SARS-CoV-2 virus may additionally affect the heart, gastrointestinal system, liver, kidney, and the central nervous system, eventually leading to multi-organ failures (Zhu et al., 2020). The disease is caused by a new strain of betacoronavirus called Severe Acute Respiratory Syndrome Coronavirus 2 (SARS-CoV-2) (Huang et al., 2020; Zhu et al., 2020; Synowiec et al., 2021). In the viral phase of the COVID-19 disease (phase I, IIa), antiviral therapy is primarily aiming to inhibit the replication of the virus or its entry into the cell (Szekanecz et al., 2021). The Spike protein expressed on the surface of the virus plays a key role in the viral entry into the host cell. The receptor-binding domain (RBD) of the Spike protein binds to the angiotensin*-*converting enzyme 2 (ACE2) receptor located on the target cell plasma membrane and facilitates host cell invasion. The use of RBD targeted (neutralizing) monoclonal antibodies is one of the therapeutic options (Majumder and Minko, 2021). To avoid resistance due to the RBD modifications observed in new SARS-CoV-2 virus variants, combinations of monoclonal antibodies, specific for different epitopes, are preferentially administered.

Recently several monoclonal antibodies have been developed to treat COVID-19. Bamlanivimab (also known as LY3819253 or LY-CoV555) and etesevimab (LY3832479 or LY-CoV016) are potent anti-spike neutralizing monoclonal antibodies, which were derived from patients recovered from COVID-19 (Jones et al., 2020; Shi et al., 2020). Plasma containing antiviral antibodies obtained from such patients has been successfully used as passive immunotherapy in a number of viral infections in the past. The use of convalescent plasma in COVID-19 patients is supported by the results, that higher plasma levels of IgG against SARS-CoV-2 was associated with lower mortality (Joyner et al., 2021). Antibodies in convalescent plasma exert their effects through neutralization of pathogens and special antibody effector mechanisms (Mair-Jenkins et al., 2015). Antibodies that inhibit the binding and entry of the virus into the cell with high specificity can be obtained from the B-lymphocytes of patients who have recovered from COVID-19 disease. These neutralizing antibodies can be modified and produced in large quantities by recombinant technology. The binding site of monoclonal antibodies is mostly the receptor binding domain (RBD) of the S protein of SARS-CoV-2 (Wu Y. et al., 2020). Bamlanivimab is an IgG1 isotype monoclonal neutralizing antibody that binds to a key region of RBD that prevents the virus from entering the cell. The antibody reportedly reduced viral load as well as viral replication in the airways. In combination with etesevimab the risk of hospitalization and death could be reduced in mild to moderate SARS-CoV-2 virus infection. It may also be used prophylactically in high-risk population following exposure to SARS-CoV-2 (Gottlieb et al., 2021).

The effector function of antibody therapeutics is greatly affected by their *N-*linked carbohydrates at the conserved Fc region, influenced by the manufacturing process. Among other techniques, various capillary gel electrophoresis methods are widely utilized in the biopharmaceutical industry for the characterization of therapeutic antibodies. For size-based protein level analysis of monoclonal antibody drugs, high-resolution separation by sodium dodecyl sulfate capillary gel electrophoresis (SDS-CGE) is commonly used, featuring rapid analysis time and low volume sample requirement (Sänger-van De Griend, 2019). To yield charge heterogeneity information of therapeutic antibodies, capillary isoelectric focusing (cIEF) is an effective CE-based separation method for amphoteric protein compounds according to their isoelectric points (pI) along a continuous pH gradient (Righetti et al., 2013). On the other hand, capillary gel electrophoresis with laser induced fluorescent detection (CGE-LIF) is the separation technique of choice for *N-*glycosylation profiling and sequencing for glycan structure elucidation after derivatization with a fluorescent label of aminopyrenetrisulfonate of PNGase F released carbohydrates (Guttman, 1996).

In this paper we introduce a capillary gel electrophoresis-based characterization workflow using the model compound of bamlanivimab, in respect to linked carbohydrate mediated size, charge and *N-*glycosylation variations. Capillary SDS gel electrophoresis was used for size heterogeneity analysis to learn about the presence/absence of the non *N-*glycosylated heavy chain (NGHC) fragment. Capillary gel isoelectric focusing was used for charge variant determination, e.g., for excess sialylation. Finally, capillary gel electrophoresis was applied for *N-*glycosylation profiling and sequencing.

## Materials and Methods

### Chemicals and Reagents

Sodium hydroxide (S2770-100ML), hydrocloric acid (1.09058.1000), sodium cyanoborohydride in 1 M tetrahydrofuran (296813-100ML), phosphoric acid (345245-100ML), urea (U0631-500G), iminodiacetic acid (220000-500G), iodoacetamide (I6125-5G) and L-arginine (A5006-100G) were purchased from Merck (Kenilworth, NJ, United States). Glacial acetic acid (02790-101-340) and acetonitrile (00630-517-350) were from Molar Chemicals (Halasztelek, Hungary). Glycerol (24388.295), tetrahydrofuran (28553.293) and 2-mercaptoethanol (M131-250ML) were from VWR (Radnor, PA, United States). The Pharmalyte 3-10 (17-0456-01) was from GE Healthcare (Chicago, IL, United States). The Advanced cIEF Starter Kit (A80976), the SDS-MW Analysis Kit (390953) and the Fast Glycan Kit (B94499PTO) were from Bio-Science Kft (Budapest, Hungary). The PNGase F enzyme and all exoglycosidase enzymes (Neuraminidase, Galactosidase and Hexosaminidase) were from the Bio-Nanosystems Laboratory, University of Pannonia (Veszprem, Hungary). The bamlanivimab (35 mg/ml; NDC 0002-7910-01) was a kind gift of the Borsod Academic County Hospital (Miskolc, Hungary).

### SDS Capillary Gel Electrophoresis

All SDS-CGE measurements were performed on a P/ACE MDQ instrument equipped with UV detection (220 ± 10 nm) (Beckman Coulter, Brea, CA, United States). The sample preparations were carried out using 80 µl of sample buffer, 5.0 µl of 2-mercaptoethanol (reduced) or 5.0 µl of 250 mM iodoacetamide (non-reduced) and 2.0 µl of 10 kDa internal standard for 10 µl of 10 mg/ml (100 µg) bamlanivimab. The samples were incubated at 70°C for 15 min prior to analysis. The instrument was equipped with a 30 cm total length (20 cm effective length, 50 μm i.d.) bare fused silica (BFS) capillary. The capillary was filled with the SDS-MW separation gel buffer and 15 kV separation voltage was applied in reversed polarity mode at 25°C capillary temperature. The sample was introduced by applying 5.0 kV for 20 s. The capillary was conditioned prior and in between every run by rinsing with 0.1 M NaOH, 0.1 M HCl and HPLC grade water, respectively.

### Isoelectric Point Determination With cIEF

Capillary gel isoelectric focusing-based isoelectric point (pI) determination was accomplished in the P/ACE MDQ instrument equipped with UV detection (280 ± 10 nm) using a 30 cm total length (20 cm effective length, 50 μm i.d.) NCHO coated capillary. The separation and sample storage temperatures were set to 20 and 10°C, respectively. The cIEF analysis workflow was carried out in two major steps: *1*) sample focusing by applying 833 V/cm electric field in normal polarity mode for 15 min between the anolyte (200 mM phosphoric acid) and catholyte (300 mM sodium hydroxide, and 2) mobilization was performed using 1000 V/cm electric field in normal polarity mode between the anolyte and the chemical mobilizer of 350 mM acetic acid for 45 min. The capillary was filled with 2.0 µl of 10 mg/ml of bamlanivimab diluted with 240 µl of Master Mix including the cIEF separation gel buffer, urea, Pharmalyte 3-10, L-arginine, iminodiacetic acid and the pI markers 7.0 and 10. The separation capillary was rinsed with 6 M urea and HPLC grade water prior and between every runs.

### *N-*Glycan Analysis and Sequencing With CGE-LIF

*N*-glycosylation analysis was also carried out using the P/ACE MDQ instrument equipped with LIF detection (ex.: 488 nm, em.: 520 nm), using a 50 cm total length (40 cm effective length, 50 μm i.d.) BFS capillary by applying 30 kV separation voltage in reversed polarity mode at 25°C capillary temperature. The column was filled with HR-NCHO separation gel buffer and the sample was injected after introducing a water plug (1.0 psi for 5.0 s) followed by the sample introduction (2.0 kV for 2.0 s). The *N*-glycan sample preparation was based on the method introduced by Reider *et al.* (Reider et al., 2018). Briefly, 10 μl of 10 mg/ml (100 µg) bamlanivimab was denatured at 80°C for 10 min using 2.0 µl of a denaturation mixture, followed by digestion with the addition of 1.0 µl of PNGase F in 20 µl of 20 mM ammonium acetate at 37°C for 2.0 h. After the digestion step, 20 µl of the labeling solution was added (6.0 mM of APTS, 100 mM of sodium cyanoborohydride in 1 M THF and 24% of acetic acid) and incubated overnight at 37°C with open lid (evaporative labeling). Prior to injection, the excess dye was removed using 20 µl of five-fold concentrated purification beads and 185 µl of acetonitrile alternately in total of four wash cycles and the APTS labeled sample was eluted by 100 µl of HPLC grade water. The samples were diluted tenfold using HPLC grade water and analyzed by the CGE-LIF system. Oligosaccharide sequencing was carried out by adding 1.0 µl of exoglycosidase enzymes from each type (Neuraminidase, Galactosidase and Hexosaminidase) to 10 µl of sample as described earlier (Szigeti and Guttman, 2017). Then, the samples were incubated at 37°C for 1.0 h. Prior to CGE-LIF analysis, 40 µl of HPLC grade water was added to the digested samples.

## Results

The capillary gel electrophoresis-based characterization workflow included size, charge and *N*-glycosylation variant analyses of the model mAb of bamlanivimab. Size and charge heterogeneities were investigated by SDS-CGE and cIEF, respectively. *N*-glycosylation variant analysis utilized capillary gel electrophoresis profiling after endoglycosidase release of the bound carbohydrates, fluorophore labeling as well as specific exoglycosydase array-based sequencing.

### Size Heterogeneity Characterization

**FIGURE 1:**
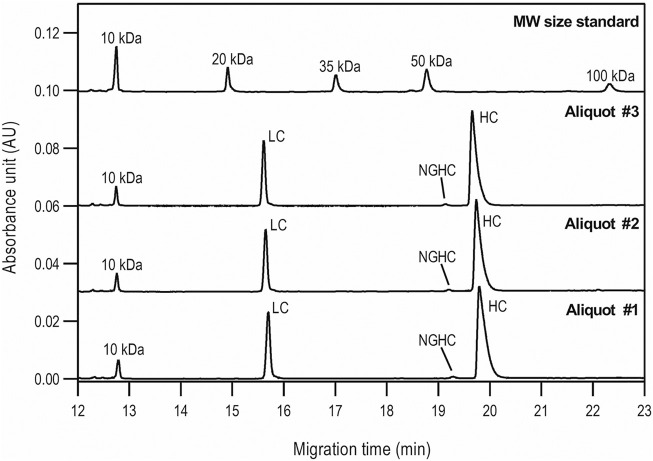
Reduced SDS-CGE analysis of bamlanivimab. Peaks: 10 kDa – internal standard, LC – light chain, NGHC – non-glycosylated heavy chain and HC - heavy chain. Separation conditions: 30 cm total capillary length, 20 cm effective length, 50 µm i.d. BFS; separation media: SDS-MW gel buffer; 25°C separation temperature; 15 kV separation voltage; sample injection: 5.0 kV for 20 s.

First, the size heterogeneity of the product was studied with special respect to the presence or absence of the NGHC fragment in reduced SDS-CGE separation modes. Purity analysis was done by SDS-CGE in non-reduced mode. In both instances, three separate aliquots of the same sample were prepared parallel and analyzed. Figure 1 compares the injections of the reduced samples to the size standard trace. The statistical analysis of the results is listed in Table 1, showing the following peak area distribution: 31.3% LC, only 0.64% NGHC and 68.06% HC fragments. Please note the excellent migration time reproducibility of <0.25% RSD.

**TABLE 1:** Statistical evaluation of the reduced SDS-CGE analysis peak profiles of bamlanivimab.

Reduced sample preparation												
Peak	Migration time (min)						%Corrected area					
	Aliquot #1	Aliquot #2	Aliquot #3	Median	Std.Dev.	%RSD	Aliquot #1	Aliquot #2	Aliquot #3	Median	Std.Dev.	%RSD
LC	15.72	15.65	15.67	15.68	0.04	0.24	31.55	31.37	30.97	31.30	0.30	0.95
NGHC	19.29	19.21	19.22	19.24	0.04	0.23	0.62	0.66	0.64	0.64	0.02	3.13
HC	19.80	19.74	19.75	19.76	0.03	0.15	67.83	67.97	68.39	68.06	0.29	0.43

**FIGURE 2:**
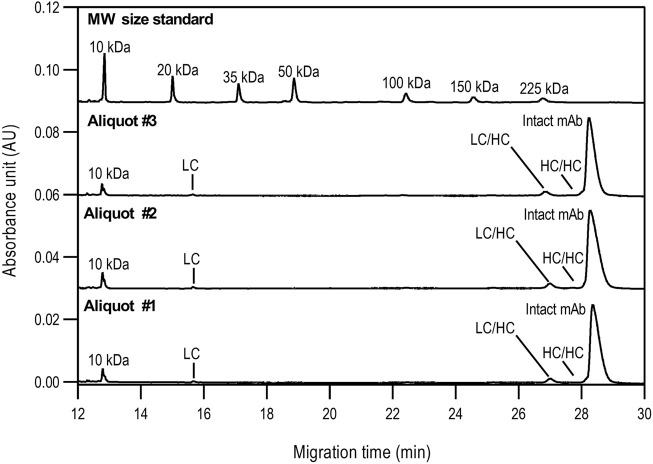
Non*-*reduced SDS-CGE analysis of bamlanivimab. Peaks: 10 kDa–internal standard, LC–light chain, LC/HC–light/heavy chain complex, HC/HC–heavy/heavy chain complex, intact mAb–intact monoclonal antibody. Separation conditions were the same as in Figure 1.

The non-reduced samples were also analyzed by SDS-CGE as shown in Figure 2, along with the statistical evaluation of the data obtained (Table 2). Similar to Figure 1, excellent migration time reproducibility was observed (<0.2% RSD) by injecting the parallel sample preparation products of the three separate aliquots. In this instance, in addition to the large intact monoclonal antibody peak (∼95.5%), some small impurity/decomposition products were also observed including the heavy-heavy chain (HC/HC) fragment, the light-heavy chain (LC/HC) fragment and the light chain (LC). The obtained purity data corresponds with the EUA FDA certificate of bamlanivimab (>95%, https://www.cellsciences.com/PDF/CPC534.pdf).

**TABLE 2:** Statistical evaluation of the non*-*reduced SDS-CGE analysis peak profiles of bamlanivimab.

Non-reduced sample preparation												
Peak	Migration time (min)						%Corrected area					
	Aliquot #1	Aliquot #2	Aliquot #3	Median	Std.Dev.	%RSD	Aliquot #1	Aliquot #2	Aliquot #3	Median	Std.Dev.	%RSD
LC	15.68	15.71	15.69	15.69	0.02	0.12	0.76	0.82	0.81	0.80	0.03	4.04
LC/HC	27.01	27.05	27.02	27.03	0.02	0.08	3.43	3.71	3.68	3.61	0.15	4.26
HC/HC	27.79	27.79	27.82	27.80	0.01	0.05	0.12	0.11	0.12	0.12	0.01	4.95
Intact mAb	28.36	28.32	28.42	28.37	0.05	0.18	95.69	95.36	95.39	95.48	0.18	0.19

### Charge Variant Analysis

**FIGURE 3:**
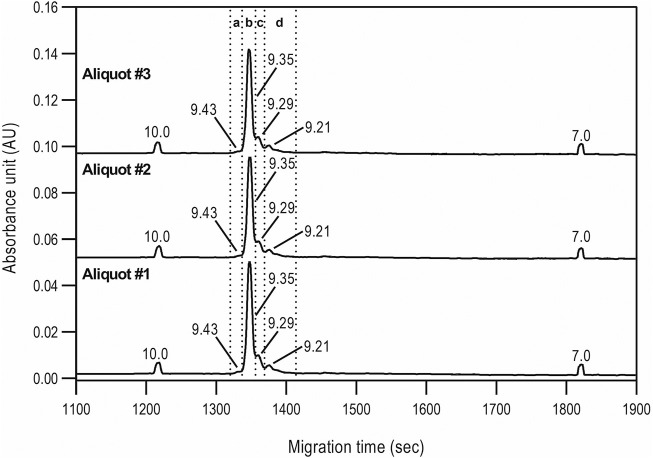
cIEF analysis of bamlanivimab. Section a: Basic peaks; Section b: Main peak; Section c: Acidic peaks region 1; Section d: Acidic peaks region 2. Separation conditions: 30 cm total capillary length, 20 cm effective length, 50 µm i.d. NCHO coated capillary; applied focusing electric field strength: 833 V/cm; mobilization electric field strength: 1,000 V/cm; 20°C separation temperature; sample injection: 2.0 µl of sample was mixed into the Master Mixture and introduced by applying 25 psi for 100 s–total capillary fill.

Capillary isoelectric focusing was used to investigate the charge variants of the product with special attention to the acidic forms assuming the presence of possible sialylated glycans. Figure 3 shows the resulting cIEF traces highlighting the basic (section a), main peak (section b), and two acidic regions (sections c and d). Very little amount (<1.5%) of basic charge variants were observed. The main peak represented 74.1% of the product. Please note that the pI difference between the main peak and the basic region was less than 0.08 pH unit. The two acidic variant regions corresponded to 13.5 and 10.8%, adding up to almost 25% as acidic forms. The very small pI differences of <0.14 pH unit suggested the need for thorough glycosylation analysis with respect to sialic acid content determination as reported below.

### *N*-Glycosylation Characterization

**FIGURE 4:**
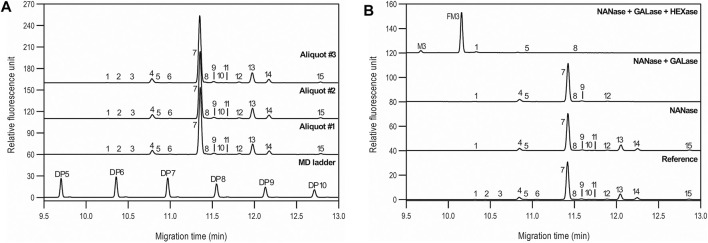
*N-*glycan profiling **(A)** and sequencing **(B)** of bamlanivimab with CGE-LIF. Peaks: as listed in Table 4. Separation conditions: 50 cm total capillary length, 40 cm effective length, 50 µm i.d. BFS; separation media: HR-NCHO gel buffer; 25°C separation temperature; 30 kV separation voltage; sample injection: 1.0 psi for 5.0 s water → 5.0 kV for 20 s sample.

The asparagine-linked glycosylation of the product was thoroughly analyzed at the profile and sequence levels by CGE as shown in Figures 4A,B, respectively. Figure 4A compares the CGE-LIF traces of the independent sample preparation products of three separate aliquots of the same sample (PNGase F digestion and APTS labeling) to the maltooligosaccharide ladder (lower trace). 14 features were identified as significant, and the peaks labeled accordingly. Glucose unit (GU) values were calculated based on the triple internal standard approach our group introduced earlier (Jarvas et al., 2016) and the corresponding structures were assigned by mining publicly available databases (glycostore.org). Table 3 lists the qualitative and quantitative results after verification by exoglycosidase array-based carbohydrate sequencing. Figure 4B compares the sequencing traces to the undigested reference sample (lower trace). Sialidase digestion (NANase trace) removed all sialic acids from the *N*-linked oligosaccharides, while consecutive galactosidase (GALase trace) and hexosaminidase (HEXase trace) treatment removed all galactose and N-acetylglucosamine residues. The large peak in trace HEXase (FM3) represents the core fucosylated trimannosyl chitobiose core structure. The total amount of core afucosylated glycans was ∼8.2%, excluding high mannose types. The galactosylated and sialylated oligosaccharides represented 17.2 and 0.5%, while 1.3% high mannose structures were detected.

**TABLE 3:** Statistical evaluation of the cIEF analysis peak profiles of bamlanivimab.

%Area							pI value						
Group	Aliquot #1	Aliquot #2	Aliquot #3	Meadian	Std.Dev.	%RSD	Peak	Aliquot #1	Aliquot #2	Aliquot #3	Meadian	Std.Dev.	%RSD
Basic peaks	1.45	1.46	1.42	1.44	0.02	1.58	1.00	9.43	9.43	9.43	9.43	0.00	0.03
Main peak	74.38	74.37	73.69	74.15	0.40	0.54	2.00	9.35	9.35	9.35	9.35	0.00	0.02
Acidic-1 peaks	13.48	13.27	13.95	13.57	0.35	2.57	3.00	9.29	9.29	9.29	9.29	0.00	0.02
Acidic-2 peaks	10.69	10.89	10.94	10.84	0.13	1.20	4.00	9.21	9.22	9.21	9.21	0.00	0.02

## Discussion

Reduced capillary SDS gel electrophoresis-based size heterogeneity analysis revealed very little amount of the non-glycosylated heavy chain fragment, suggesting that the product was >99% glycosylated (Table 1). Applying the same method to the non-reduced sample showed 95.5% purity (Table 2). Please note that while the MW of the intact form of the molecule is ∼150 kDa, it migrated slower than the 225 kDa size standard. The reason for this anomaly is the significantly bulkier Y shape of the intact IgG molecule compared to the linear polypeptide standard, as was reported earlier (Guttman et al., 2021). Charge variant characterization by capillary gel isoelectric focusing showed the presence of lower than 1.5% of the basic variants, but almost 25% of acidic variants (Table 3). However, the very small pI difference between the main peak and even the higher acidity variant region of <0.14 pH unit, suggested the need for additional glycosylation analysis with special respect to sialic acid containing attached glycans. *N*-glycosylation profiling and sequencing revealed that the total sialylation level of the product was only ∼0.5% (Table 4), therefore the acidic variants found during the cIEF analysis were probably the result of the number of acidic amino acids in the polypeptide backbone and possible deamidation or glycation of the product. The core fucosylation level of bamlanivimab was >90%, suggesting some antibody dependent cell mediated cytotoxicity (ADCC) effector function of the product due to the presence of approximately 8.2% afucosylated attached oligosaccharides. As a matter of fact, ADCC function of bamlanivimab was reported on FCγRIIIa expressing Jurkat cells after reacting with Spike protein expressing target cells, while complement dependent cytotoxicity (CDC) was not detected (Eli Lilly and Company, U.S. Food and Drug Administration, 2021). The interaction between the FcγRIIIa glycosylation and the lack of core fucosylation on the IgG Fc region enables greater carbohydrate-carbohydrate interactions increasing the overall binding strength (Hsieh et al., 2017; Wang et al., 2018). In addition, approximately 17% of the core fucosylated biantennary structures showed terminal galactosylation, reportedly enhancing the above suggested ADCC function (Raju and Jordan, 2012; Aoyama et al., 2019). Please note that terminal galactosylation also increases the serum half-life of the product, while the very low amount of high mannose structures found in our study (1.28%) should not significantly contribute to the opposite (Filep et al., 2021). By all means, our results suggested some ADCC function of bamlanivimab, besides the neutralizing effect of the product.

**TABLE 4:** Statistical evaluation of the *N-*glycan analysis peak profiles of bamlanivimab.

Peak	ID	GU	Exoglycosidase sequencing - response			Migration time (min)						%Area					
			NANase	GALase	HEXase	Aliquot #1	Aliquot #2	Aliquot #3	Median	Std.Dev.	%RSD	Aliquot #1	Aliquot #2	Aliquot #3	Median	Std.Dev.	%RSD
1	M4	5.74	—	—	—	10.28	10.26	10.25	10.26	0.01	0.13	0.19	0.19	0.19	0.19	0.00	2.17
2	FA2G1S1	5.92	X	—	—	10.40	10.38	10.38	10.39	0.01	0.12	0.13	0.13	0.13	0.13	0.00	1.26
3	A2G2S1	6.18	x	—	—	10.57	10.55	10.55	10.56	0.01	0.12	0.12	0.11	0.11	0.11	0.00	4.37
4	A2	6.56	—	—	x	10.81	10.79	10.78	10.79	0.01	0.12	4.71	4.81	4.86	4.79	0.08	1.64
5	M5	6.67	—	—	—	10.87	10.85	10.85	10.85	0.01	0.12	0.41	0.42	0.43	0.42	0.01	2.81
6	A2B	6.89	x	—	—	11.02	11.00	10.99	11.00	0.01	0.13	0.32	0.32	0.29	0.31	0.02	5.52
7	FA2	7.49	—	—	x	11.38	11.35	11.35	11.36	0.01	0.12	73.51	73.32	73.74	73.52	0.21	0.28
8	M6	7.59	—	—	—	11.44	11.42	11.42	11.42	0.01	0.09	0.70	0.66	0.67	0.68	0.02	2.63
9	N/A	7.76	—	—	x	11.54	11.52	11.51	11.52	0.01	0.12	1.65	1.71	1.64	1.66	0.04	2.26
10	A2G1	7.90	—	x	—	11.63	11.61	11.61	11.61	0.01	0.11	0.23	0.23	0.21	0.23	0.01	5.19
11	A2B[6]G1	8.01	—	x	—	11.69	11.67	11.67	11.68	0.01	0.12	0.23	0.24	0.23	0.23	0.00	1.45
12	N/A	8.26	—	—	x	11.84	11.82	11.82	11.83	0.01	0.12	0.94	0.95	0.92	0.93	0.02	1.84
13	FA2[6]G1	8.53	—	x	—	12.00	11.98	11.97	11.98	0.01	0.11	11.67	11.73	11.55	11.65	0.10	0.82
14	FA2[3]G1	8.85	—	x	—	12.20	12.17	12.17	12.18	0.01	0.12	4.22	4.21	4.12	4.18	0.05	1.24
15	FA2G2	9.87	—	x	—	12.81	12.78	12.78	12.79	0.01	0.11	0.99	0.98	0.93	0.96	0.03	3.40
Total afucosylated (incl. high mannose) peaks (%)					9.56	%RSD median - all peaks (>0.01%)					0.12	%RSD median - all peaks (>0.01%)					2.46
Total terminal galactosylated peaks (%)					17.25	%RSD median - %area below 1%					0.12	%RSD median - %area below 1%					3.06
Total high mannose peaks (%)					1.29	%RSD median - %area between 1 and 5%					0.12	%RSD median - %area between 1 and 5%					1.71
Total sialylated peaks (%)					0.55	%RSD median - %area over 5%					0.12	%RSD median - %area over 5%					0.55

## Conclusion

In this paper, a comprehensive capillary gel electrophoresis-based characterization workflow is presented using bamlanivimab, an anti-COVID-19 biotherapeutic product, as a model compound. The study included analyses at the intact protein, subunit and *N*-glycosylation levels using capillary SDS gel electrophoresis and isoelectric focusing with UV detection as well as GGE-LIF, respectively. This latter was utilized for profiling and sequencing to reveal possible *N*-glycan structure–function relationships. Our results revealed the presence of negligible amount of non glycosylated HC fragment, significant amount of acidic charge variants (25%) but with very little pI shift (<0.14 pH unit) and >90% of fucosylated *N*-linked oligosaccharides. The remaining <10% core-afucosylated glycans together with the 17% terminal galactosylation suggested some ADCC effector function of the product, while the major mode of action of bamlanivimab should still be considered by its neutralizing effect.

## Data Availability

The raw data supporting the conclusions of this article will be made available by the authors, without undue reservation.

## Abbreviations

ADCC, antibody dependent cell mediated cytotoxicity; CGE-LIF, capillary gel electrophoresis with laser induced fluorescent detection; cIEF, capillary isoelectric focusing; HC, IgG heavy chain; LC, IgG light chain; NGHC, non-glycosylated IgG heavy chain; pI, isoelectric point; PNGase F, Peptide-N4-(N-acetyl-beta-glucosaminyl) asparagine amidase; RBD, receptor binding domain; SDS-CGE, sodium dodecyl sulfate – capillary gel electrophoresis.
